# Supplementary material for: A maternal brain hormone that builds bone
Source: Nature. 2024 Jul 10;632(8024):357–65. doi: 10.1038/s41586-024-07634-3 (PMC11306098; doi:10.1038/s41586-024-07634-3)
Supplement: Supplementary file 2 — Reporting Summary [file 41586_2024_7634_MOESM2_ESM.pdf]

nature portfolio

Corresponding author(s): Dr. Holly Ingraham and Dr. Thomas Ambrosi

Last updated by author(s): June 26, 2024

Reporting Summary

Nature Portfolio wishes to improve the reproducibility of the work that we publish. This form provides structure for consistency and transparency in reporting. For further information on Nature Portfolio policies, see our Editorial Policies and the Editorial Policy Checklist.

Statistics

For all statistical analyses, confirm that the following items are present in the figure legend, table legend, main text, or Methods section.

n/a	Confirmed	
☐	☒	The exact sample size (*n*) for each experimental group/condition, given as a discrete number and unit of measurement
☐	☒	A statement on whether measurements were taken from distinct samples or whether the same sample was measured repeatedly
☐	☒	The statistical test(s) used AND whether they are one- or two-sided<br>*Only common tests should be described solely by name; describe more complex techniques in the Methods section.*
☐	☒	A description of all covariates tested
☐	☒	A description of any assumptions or corrections, such as tests of normality and adjustment for multiple comparisons
☐	☒	A full description of the statistical parameters including central tendency (e.g. means) or other basic estimates (e.g. regression coefficient) AND variation (e.g. standard deviation) or associated estimates of uncertainty (e.g. confidence intervals)
☐	☒	For null hypothesis testing, the test statistic (e.g. *F*, *t*, *r*) with confidence intervals, effect sizes, degrees of freedom and *P* value noted<br>*Give P values as exact values whenever suitable.*
☒	☐	For Bayesian analysis, information on the choice of priors and Markov chain Monte Carlo settings
☒	☐	For hierarchical and complex designs, identification of the appropriate level for tests and full reporting of outcomes
☒	☐	Estimates of effect sizes (e.g. Cohen's *d*, Pearson's *r*), indicating how they were calculated

Our web collection on statistics for biologists contains articles on many of the points above.

Software and code

Policy information about availability of computer code

Data collection	qPCR data was acquired using the CFX Maestro software (Bio-Rad, v4.1.2433.1219). Bulk RNA-seq reads were sequenced on Illumina's NovaSeq 6000, S4 flow cell. For histomorphometry, mounted sections were imaged with an ECHO REVOLVE R4 using FITC (Calcein) and TexasRed (Alizarin Red) channels by the Wash U Bone Core. Mosaic-tiled images of distal femurs were acquired at ×20 magnification with a Zeiss Axioplan Imager M1 microscope (Carl Zeiss MicroImaging) fitted with a motorized stage. The tiled images were stitched and converted to a single image using the Axiovision software (Carl Zeiss MicroImaGing). For SSCs MBH brain transplants, images of histological sections were taken using the Keyence B2-X800. For kidney transplants, brightfield images were taken using a Luminera Infinity-3 and quantified using ImageJ software. In vivo micro (µ) CT was performed using a Scanco Viva CT40 high-speed µCT preclinical scanner. For ex-vivo µCT, a Scanco Medical µCT 50 specimen scanner calibrated to a hydroxyapatite phantom or Bruker SkyScan1276 (Bruker Preclinical Imaging) was utilized. Standard best practices were used to quantify trabecular and cortical bone parameters44. Image acquisition of an implanted femur was captured with iPhone 13 Pro. Body composition to determine % lean and fat mass was obtained by dual-energy x-ray (DEXA, GE Lunar PIXImus). Femurs underwent a three-point bend test using the Instron E100 mechanical load frame. For brain RNAscope and immunohistochemistry, confocal images were acquired at the UCSF Nikon Imaging Center using a Nikon CSU-22 with an EMCCD camera and MicroManager v2.0gamma. for single-cell RNA-sequencing of ocSSCs, pooled libraries were sequenced on NovaSeq6000 (Illumina) to obtain 1–2 million 2 x 151 base-pair paired-end reads per cell. Flow cytometry and cell sorting were performed on a FACS Aria II cell sorter (BD Biosciences).
Data analysis	For histomorphometry, photoshop software removed the background in non-tissue areas for images of the proximal tibias. Blinded analyses were performed using two image-analysis software programs, Bioquant OSTEO software (Nashville, TN, USA) or ImageJ software. Images taken with iPhone 13 Pro were edited in Photoshop CC. For HFD experiment, Volumes of interest were evaluated using Scanco evaluation software. Representative 3D images were created using Scanco Medical mCT Ray v4.0 software. For the whole femur bone cultures, naloxone and S961 experiments, ccnn3 injections into WT mice, GOF CCN3 hepatic expression and LOF Ccn3 knowchdown in ARC, reconstructed

samples were analyzed using CT Analyser and CTvox software (Bruker). Confocal images were processed and quantified using ImageJ FIJI v1.52i and the Cell Counter plugin v2. For all bulk RNAseq samples, sequencing-generated reads were aligned to the mouse transcriptome (mm10) using Kallisto in gene mode47. Differential gene expression was evaluated using the likelihood-ratio test by Sleuth (qval <0.05)48. All heatmaps were generated with the top 50 female/male-biased genes obtained from 27-week-old mice and were generated in R49. For single cell RNAseq sequencing, sequenced data were demultiplexed using bcl2fastq2 2.18 (Illumina). Raw reads were further processed using a skewer for 3′ quality trimming, 3′ adaptor trimming, and removal of degenerate reads. Trimmed reads were then mapped to the mouse genome vM20 using STAR 2.4, and counts for gene and transcript reads were calculated using RSEM 1.2.21. Data were explored, and plots were generated using Scanpy v1.9. To select high-quality cells only, we excluded cells with fewer than 450 genes and genes detected in less than three cells were excluded. Cells with a mitochondrial gene content higher than 5%, ERCC content higher than 30%, and ribosomal gene content higher than 5% were excluded as well. Scrublet was then used to detect and remove residual duplicates. A total of 264 high-quality cells (122 control and 142 mutant mouse cells) were included in the final analysis. Raw counts per million (CPM) values were mean- and log-normalized, and then data were scaled to a maximum value of 10. Combat batch correction was applied to account for potential biases through minor differences in cell processing. Principal component (PC) 'elbow' heuristics were used to determine the number of PCs for clustering analysis with UMAP and Leiden Algorithm (leidenalg). Differential gene expression between Esr1fl/fl (wild type) and Esr1Nkx2-1Cre (mutant), as well as Leiden clusters, was calculated by the Wilcoxon-Rank-Sum test. Cell cycle status was assessed using the 'score_genes_cell_cycle' function with the updated gene list provided by Nestorowa et al.51. EnrichR was used to explore enrichment for pathways and ontologies of differentially expressed genes between wild-type and mutant groups52. Statistical tests, excluding RNA-Seq analyses, were performed using Prism 10 (GraphPad). Flow cytometry and cell sorting were analyzed using FlowJo software.

For manuscripts utilizing custom algorithms or software that are central to the research but not yet described in published literature, software must be made available to editors and reviewers. We strongly encourage code deposition in a community repository (e.g. GitHub). See the Nature Portfolio guidelines for submitting code & software for further information.

Data

Policy information about availability of data

All manuscripts must include a data availability statement. This statement should provide the following information, where applicable:

- Accession codes, unique identifiers, or web links for publicly available datasets
- A description of any restrictions on data availability
- For clinical datasets or third party data, please ensure that the statement adheres to our policy

A data availability statement is included in the manuscript. All data generated or analyzed during this study will be included in the published article (and its supplementary information files). All raw data and processed data files for the bulk-RNA and sc-RNA Sequencing are publicly available at GEO under sample accession numbers GSE248882 and GSE241478.

Research involving human participants, their data, or biological material

Policy information about studies with human participants or human data. See also policy information about sex, gender (identity/presentation), and sexual orientation and race, ethnicity and racism.

Reporting on sex and gender	Male and Female
Reporting on race, ethnicity, or other socially relevant groupings	NA
Population characteristics	Different Age Groups and Different Biological Sex
Recruitment	NA
Ethics oversight	UC Davis IRB ID: 1997852-3, waived

Note that full information on the approval of the study protocol must also be provided in the manuscript.

Field-specific reporting

Please select the one below that is the best fit for your research. If you are not sure, read the appropriate sections before making your selection.

☒ Life sciences ☐ Behavioural & social sciences ☐ Ecological, evolutionary & environmental sciences

For a reference copy of the document with all sections, see nature.com/documents/nr-reporting-summary-flat.pdf

Life sciences study design

All studies must disclose on these points even when the disclosure is negative.

Sample size	Sample sizes were based on previous work from our lab and others (Correa et al., 2015, Herber et al., 2019, van Veen et al,. 2020, Ambrosi et al., 2021). For other studies statistical calculation was performed to determine sample size using open source software (G-power etc).
Data exclusions	Animals that unexpectedly became morbid during the course of parabiosis were excluded.

Data exclusions	Only animals that survived to final timepoints of experiments were included. During processing of single cell genomic data filtering of low quality cells was applied as described.
Replication	All data presented are biological replicates unless otherwise stated in the figure legends. Each experimental finding was reproduced in at least two independent experiments or at different concentrations, with the exception of parabiosis.
Randomization	Mice were drawn at random from a pool of littermate mice containing a roughly equal mix of Cre+ and Cregenotypes. Partitioning into control and experimental groups was determined by genotype. For experiments involving repeated measurements, a randomized balanced design was implemented such that a mix of control and experimental mice housed in the same cage received identical treatments during each trial.
Blinding	Measurements of micro CT bone parameters, osmium stain, histology, histomorphometry, DEXA, qPCR, bulk/single RNA-Seq data and glucose tolerance test and plasma triglyceride assay were made by experimenters blinded to the genotype and treatment.

Reporting for specific materials, systems and methods

We require information from authors about some types of materials, experimental systems and methods used in many studies. Here, indicate whether each material, system or method listed is relevant to your study. If you are not sure if a list item applies to your research, read the appropriate section before selecting a response.

Materials & experimental systems

n/a	Involved in the study
☐	☒ Antibodies
☒	☐ Eukaryotic cell lines
☒	☐ Palaeontology and archaeology
☐	☒ Animals and other organisms
☒	☐ Clinical data
☒	☐ Dual use research of concern
☒	☐ Plants

Methods

n/a	Involved in the study
☒	☐ ChIP-seq
☐	☒ Flow cytometry
☒	☐ MRI-based neuroimaging

Antibodies

Antibodies used	ERa (EMD Millipore, #C1355 polyclonal rabbit, 1:750 dilution). Kiss1 (Abcam, #ab19028 polyclonal rabbit, 1:200 dilution). CCN3 (R&D Systems, #AF1976 polyclonal goat, 1:1000 dilution). HRP-conjugated secondary antibody for CCN3 (Invitrogen, ##A15999, 1:30000).Species-appropriate secondary Alexa Fluor-coupled antibodies (Invitrogen, #A-21447, #A10042, or #A-11055; 1:1000 dilution). For mouse SSC lineages, antibodies used were as follows: CD90.1 (Thermo Fisher, Cat# 47–0900), CD90.2 (Thermo Fisher, Cat#47–0902), CD105 (Thermo Fisher, Cat#13–1051), CD51 (BD Biosciences, Cat#551187), CD200 (Thermo Fisher Cat#MA5-17980), CD45 (BioLegend, Cat#103110), Ter119 (Thermo Fisher, Cat#15–5921), Tie2 (Thermo Fisher, Cat#14–5987) 6C3 (BioLegend, Cat#108312), and Streptavidin PE-Cy7 (Thermo Fisher, Cat#25–4317) as well as Sca-1 (Thermo Fisher, Cat#56-5981), CD45 (Thermo Fisher, Cat#11–0451), CD31 (Thermo Fisher, Cat#12–0311), CD140a (ThermoFisher, Cat#17–1401), CD24 (Thermo Fisher, Cat#47–0242). For human SSC isolation, antibodies used were as follows: CD45 (BioLegend, Cat#304029), CD235a (BioLegend, Cat#306612), CD31 (Thermo Fisher Scientific, Cat#13-0319), CD202b (TIE-2) (BioLegend, Cat#334204), streptavidin APC-AlexaFlour750 (Thermo Fisher, SA1027), CD146 (BioLegend, Cat#342010), PDPN (Thermo Fisher Scientific, Cat#17-9381), CD164 (BioLegend, Cat#324808), and CD73 (BioLegend, Cat#344016).
Validation	ERalpha antibody validated by the manufacturer to detect ERalpha in breast cancer cell lines. In addition hypothalamic ERalpha expression was absent in conditional ERalpha knockout mice.<br>Kiss 1 antibody validated by the manufacturer to dected Kiss 1. See https://www.abcam.com/kisspeptin-antibody-ab19028-references.html#top-608.<br>CCN3 antibody validated by the manufacturer to detect CCN3 in conditioned medium of Sf9y82 cells infected with a recom-binant baculovirus expressing CCN3 (NOV) in the sense orientation. Perbal, B. et al. (1999) Proc. Natl. Acad. Sci. USA 96:869.<br>All antibodies were used for flow cytometry and are validated, commercially available products that addtionally have been validated in previously published studies (e.g. PMID: 29748647 & PMID: 15967997).<br>FACS Sorting Antibodies - Mouse Listed Below All antibodies were used for flow cytometry and are validated, commercially available products that addtionally have been validated in previously published studies (e.g. PMID: 29748647 & PMID: 15967997).<br>Anti-mouse Thy1.1 Cat#: 47-0900), CD90.1 (Thy-1.1) Monoclonal Antibody (HIS51), APC-eFluor 780, eBioscience™ Reported for flow cytometry recognizing mouse CD90.1<br>Anti-mouse Thy1.2 (Cat#: 47-0902), CD90.2 (Thy-1.2) Monoclonal Antibody (53-2.1), APC-eFluor 780, eBioscience™ Reported for flow cytometry recognizing mouse CD90.2<br>Anti-mouse CD105 Cat#: 13-1051), CD105 (Endoglin) Monoclonal Antibody (MJ7/18), Biotin, eBioscience™ Reported for flow cytometry recognizing mouse CD105<br>Anti-mouse CD51 Cat#: 551187), CD51 (Integrin alpha V) Monoclonal Antibody (RMV-7), PE, BD Biosciences, Reported for flow cytometry recognizing mouse CD51<br>Anti-mouse CD200 Cat#: MA5-17980), CD200 Monoclonal Antibody (OX90), FITC, eBioscience™ Reported for flow cytometry recognizing mouse CD200<br>Anti-mouse Ter119 Cat#: 15-5921), TER-119 Monoclonal Antibody (TER-119), PE-Cyanine5, eBioscience™ Reported for flow cytometry recognizing mouse Ter119<br>Anti-mouse Tie2 Cat#: 14-5987), CD202b (TIE2) Monoclonal Antibody (TEK4), eBioscience™ Reported for flow cytometry recognizing mouse Tie2

Anti-mouse 6C3 Cat#: 108312), Ly-51 Antibody Monoclonal Antibody (6C3), Alexa Fluor® 647, BioLegend Reported for flow cytometry recognizing mouse CD249
Anti-streptavidin-PE-Cy7 Cat# 25–4317), eBioscience™ Streptavidin PE-Cyanine7 Conjugate Reported for flow cytometry recognizing Biotin
Anti-mouse Sca1 Cat# 56-5981), Ly-6A/E (Sca-1) Monoclonal Antibody (D7), Alexa Fluor™ 700, eBioscience™ Reported for flow cytometry recognizing mouse Ly-6A/E
Anti-mouse CD45 Cat#: 103110), CD45 Monoclonal Antibody (30-F11), PE-Cyanine5, BioLegend Reported for flow cytometry recognizing mouse CD45
Anti-mouse CD45 Cat#: 11–0451), CD45 Monoclonal Antibody (30-F11), FITC, eBioscience™ Reported for flow cytometry recognizing mouse CD45
Anti-mouse CD31 Cat#: 12-0311), CD31 Monoclonal Antibody (390), PE, eBioscience™ Reported for flow cytometry recognizing mouse CD31
Anti- mouse CD140a Cat#17–1401), CD140a Monoclonal Antibody (APA5), eBioscience™ Reported for flow cytometry recognizing mouse CD140a
Anti-mouse CD24 Cat#47–0242), CD24 Monoclonal Antibody (M1/69), APC-eFluor780, eBioscience™ Reported for flow cytometry recognizing mouse CD24

FACS Sorting Antibodies -Human Listed Below All antibodies were used for flow cytometry and are validated, commercially available products that addtionally have been validated in previously published studies (e.g. PMID: 29748647 & PMID: 15967997).
Mouse anti-human CD45 Cat#304029), CD45 Monoclonal Antibody (HI30), Pacific Blue™,, BioLegend Reported for flow cytometry recognizing anti-human CD45
Mouse anti-human CD235ab Cat#306612), CD235ab Monoclonal Antibody (HIR2), Pacific Blue™, BioLegend Reported for flow cytometry recognizing anti-human CD235a
Mouse anti-human CD31 Cat#13-0319), CD31 Monoclonal Antibody (WM-59), Biotin, eBioscience™ Reported for flow cytometry recognizing anti-human CD31
Anti-streptavidin-APC-AlexaFlour750 Cat#SA1027), Streptavidin, (APC-Alexa Fluor™ 750), Thermo Fisher Reported for flow cytometry recognizing Biotin
Mouse anti-human CD202b Cat#334204), CD202b Monoclonal Antibody CD202b (33.1), Biotin-linked, BioLegend Reported for flow cytometry recognizing anti-human CD202B
Mouse anti-human CD146 at#342010), CD46 Monoclonal Antibody (SHM-57), PE-Cy7, Biolegend Reported for flow cytometry recognizing anti-human CD146
Mouse anti-human PDPN Cat#17-9381), PDPN Monoclonal Antibody (NZ 1.3), APC, Thermo Fisher Reported for flow cytometry recognizing anti-human PDPN
Mouse anti-human CD164 Cat#324808), CD 164 Monoclonal Antibody (67D2), PE, BioLegend Reported for flow cytometry recognizing anti-human CD164
Mouse anti-human CD73 Cat#344016), CD73 Monoclonal Antibody (Ad2), FITC, BioLegend Reported for flow cytometry recognizing anti-human CD73

Animals and other research organisms

Policy information about studies involving animals; ARRIVE guidelines recommended for reporting animal research, and Sex and Gender in Research

Laboratory animals	The origin of the Esr1fl/fl allele (official allele: Esr1tm1Sakh) on a 129P2 background and used to generate Esr1Nkx2-1Cre mice are described1 and were maintained on CD-1;129P2 mixed background. Primer sequences used for genotyping are listed in Extended Data Table 1. Esr1Nkx2-1Cre-CAG-Luc,-GFP mice were generated by crossing male mice harboring the CAG-Luc-GFP allele (official allele: L2G85Chco/J) to female mice homozygous for the Esr1fl/fl allele, followed by an additional cross to generate Esr1fl/fl; Nkx2-1Cre; Luc-GFP colony, which was maintained on a mixed FVB/N, CD-1, 129P2, and C57BL/6 genetic background. Esr1ProdynorphinCre mice were generated by crossing homozygous Esr1fl/fl females to Prodynorphin-Cre (B6;129S-Pdyntm1.1(cre)Mjkr/LowlJ, purchased from JAX) males. Unless otherwise noted, mice were maintained on a 12 h light/dark cycle with ad libitum access to a standard breeder chow diet (PicoLab 5058; LabDiet, 4kcal% fat, 0.8% Ca2+) and sterile water and housed under controlled and monitored rooms for temperature and humidity with a 12h light/dark cycle. Eighteen-month-old C57BL/6-aged female mice were obtained through the NIA Aged Rodent Colony Program, available to NIA Funded Projects. To create cohorts of ovariectomized (OVX) females, ovariectomy was performed at 4 months of age, followed by 4 weeks of surgical recovery. All animal procedures were performed in accordance with UCSF institutional guidelines under the Ingraham lab IACUC protocol of record.
Wild animals	No wild animals used.
Reporting on sex	In all studies, sex as a biological factor was considered and for nearly all analyses unless indicated both male and female mice were used.

Field-collected samples | No samples collected from the field.

Ethics oversight | Experiments were approved and performed in accordance with the guidelines of the UCSF Institutional Animal Care Committee (IACUC) or the UCD Animal Ethics Committee, the National Institutes of Health Guide for Care and Use of Laboratory Animals, and recommendations of the International Association for the Study of Pain.

Note that full information on the approval of the study protocol must also be provided in the manuscript.

Flow Cytometry

Plots

Confirm that:

- [ ] The axis labels state the marker and fluorochrome used (e.g. CD4-FITC).
- [ ] The axis scales are clearly visible. Include numbers along axes only for bottom left plot of group (a 'group' is an analysis of identical markers).
- [ ] All plots are contour plots with outliers or pseudocolor plots.
- [ ] A numerical value for number of cells or percentage (with statistics) is provided.

Methodology

Sample preparation | Detailed sample preparation protocol is provided in methods section of the manuscript (PMID: 29748647 & PMID: 15967997).

Instrument | Flow cytometry was performed on FACS Aria II (BD Biosciences).
Gating schemes were established with fluorescence-minus-one (FMO: staining with all fluorophores except one) controls and negative propidium iodide (PI) (Sigma-Aldrich, Cat#P4170) staining (1 mg/ml) was used as a measure for cell viability.

Software | FlowJo v10 was used to analyze FACS data.

Cell population abundance | Quantification of cell populations are provided as total number of tissue or percentage of reference population as described in the figure and figure legends.

Gating strategy | Gating Strategy is provided in previously published manuscripts (PMID: 29748647 & PMID: 15967997).

- [ ] Tick this box to confirm that a figure exemplifying the gating strategy is provided in the Supplementary Information.
